# Socio-economic and demographic determinants of female genital mutilation in sub-Saharan Africa: analysis of data from demographic and health surveys

**DOI:** 10.1186/s12978-020-01015-5

**Published:** 2020-10-22

**Authors:** Bright Opoku Ahinkorah, John Elvis Hagan, Edward Kwabena Ameyaw, Abdul-Aziz Seidu, Eugene Budu, Francis Sambah, Sanni Yaya, Eric Torgbenu, Thomas Schack

**Affiliations:** 1 School of Public Health, Faculty of Health, University of Technology Sydney, Sydney, Australia; 2 Department of Health, Physical Education, and Recreation, University of Cape Coast, Cape Coast, Ghana; 3 Neurocognition and Action-Biomechanics-Research Group, Faculty of Psychology and Sport Sciences, Bielefeld University, Bielefeld, Germany; 4 Department of Population and Health, University of Cape Coast, Cape Coast, Ghana; 5 College of Public Health, Medical and Veterinary Sciences, James Cook University, Townsville, QLD Australia; 6 School of International Development and Global Studies, University of Ottawa, Ottawa, Canada; 7 The George Institute for Global Health, The University of Oxford, Oxford, UK; 8 Department of Physiotherapy and Rehabilitation Sciences, School of Allied Health Sciences, University of Health and Allied Sciences, Ho, Ghana

**Keywords:** FGM, Public health, Socioeconomic, Women, Daughters, SSA

## Abstract

**Background:**

Owing to the severe repercussions associated with female genital mutilation (FGM) and its illicit status in many countries, the WHO, human rights organisations and governments of most sub-Saharan African countries have garnered concerted efforts to end the practice. This study examined the socioeconomic and demographic factors associated with FGM among women and their daughters in sub-Saharan Africa (SSA).

**Methods:**

We used pooled data from current Demographic and Health Surveys (DHS) conducted between January 1, 2010 and December 31, 2018 in 12 countries in SSA. In this study, two different samples were considered. The first sample was made up of women aged 15–49 who responded to questions on whether they had undergone FGM. The second sample was made up of women aged 15–49 who had at least one daughter and responded to questions on whether their daughter(s) had undergone FGM. Both bivariate and multivariable analyses were performed using STATA version 13.0.

**Results:**

The results showed that FGM among women and their daughters are significantly associated with household wealth index, with women in the richest wealth quintile (AOR, 0.51 CI 0.48–0.55) and their daughters (AOR, 0.64 CI 0.59–0.70) less likely to undergo FGM compared to those in the poorest wealth quintile. Across education, the odds of women and their daughters undergoing FGM decreased with increasing level of education as women with higher level of education had the lowest propensity of undergoing FGM (AOR, 0.62 CI 0.57–0.68) as well as their daughters (AOR, 0.32 CI 0.24–0.38). FGM among women and their daughters increased with age, with women aged 45–49 (AOR = 1.85, CI 1.73–1.99) and their daughters (AOR = 12.61, CI 10.86–14.64) more likely to undergo FGM. Whiles women in rural areas were less likely to undergo FGM (AOR = 0.81, CI 0.78–0.84), their daughters were more likely to undergo FGM (AOR = 1.09, CI 1.03–1.15). Married women (AOR = 1.67, CI 1.59–1.75) and their daughters (AOR = 8.24, CI 6.88–9.87) had the highest odds of undergoing FGM.

**Conclusion:**

Based on the findings, there is the need to implement multifaceted interventions such as advocacy and educational strategies like focus group discussions, peer teaching, mentor–mentee programmes at both national and community levels in countries in SSA where FGM is practiced. Other legislative instruments, women capacity-building (e.g., entrepreneurial training), media advocacy and community dialogue could help address the challenges associated with FGM. Future studies could consider the determinants of intention to discontinue or continue the practice using more accurate measures in countries identified with low to high FGM prevalence.

## Plain English summary

Owing to the severe repercussions associated with female genital mutilation (FGM) and its illicit status in many countries, the WHO, human rights organisations and governments of most sub-Saharan African countries have garnered concerted efforts to end the practice. This study examined the socioeconomic and demographic factors associated with FGM among women and their daughters in selected sub-Saharan African countries. The study used pooled data from current DHS conducted between January 1, 2010 and December 31, 2018 in 12 countries in sub-Saharan Africa. The results showed that FGM among women and their daughters decreases with wealth status, with women with the richest wealth quintile and their daughters less likely to undergo FGM compared to those with the poorest wealth quintile. Across education, the odds of women and their daughters undergoing FGM decreased with increasing level of education as women with higher level of education had the lowest propensity of undergoing FGM as well as their daughters. FGM among women and their daughters increased with age, with women aged 45–49 and their daughters more likely to undergo FGM. Whiles women in rural areas were less likely to undergo FGM, their daughters were more likely to undergo FGM. Married women and their daughters had the highest odds of undergoing FGM. Based on the findings, multifaceted interventions should include considerable efforts directed on advocacy and educational strategies like focus group discussions, peer teaching, mentor–mentee programmes at both national and community levels in regions noted with the FGM practice. Other legislative instruments, women capacity-building (e.g., entrepreneurial training), media advocacy and community dialogue could help address the FGM public health challenge.

## Background

Female genital mutilation (FGM), otherwise known as female circumcision (FC) and female genital cutting (FGC), transpire in different societies in various forms [1]. The WHO conceives FGM to “involve the partial or total removal of external female genitalia or other injury to the female genital organs for non-medical reasons” [2]. According to the WHO [3], there are 4 main types of FGM, Type 1 involves the partial or total removal of the clitoral glans (the external and visible part of the clitoris, which is a sensitive part of the female genitals), and/or the prepuce/ clitoral hood (the fold of skin surrounding the clitoral glans). Type 2 is the partial or total removal of the clitoral glans and the labia minora (the inner folds of the vulva), with or without removal of the labia majora (the outer folds of skin of the vulva). Type 3 is the most extreme type of the practice as categorized by the WHO and involves the lessening of the vaginal hole by creating a covering seal. The seal is formed by cutting and repositioning the labia minora, or labia majora, sometimes through stitching, with or without removal of the clitoral prepuce/clitoral hood and glans (Type I FGM), whereas the Type 4 includes all other harmful procedures to the female genitalia for non-medical purposes, e.g. pricking, piercing, incising, scraping and cauterizing the genital area [3]. FGM has no health benefits. The rationale for performing FGM differ from location to location, however, sociocultural factors are remarkable drivers of the practice [2]. Predominantly, it is usually conducted on young females between infancy and adolescence, and infrequently on women [2]. At least, 3 million girls are estimated to be at risk of FGM yearly [2, 4]. Over 200 million girls and women have experienced the act which occurs in more than 40 countries globally [2, 4].

FGM is practiced in about 28 countries in Africa, with most of these countries concentrated in the sub-Saharan African region [5]. FGM has thrived in sub-Saharan Africa (SSA) owing to strong socio-cultural drivers, which facilitate clandestine perpetration of the act and underreporting [6]. FGM inflicts numerous complications on its victims on the account of limited surgical competencies of most FGM practitioners (e.g. traditional birth attendants), non-utilisation of anaesthetic agents and nonexistence of antiseptic techniques [6]. These complications occur during the FGM process or shortly afterwards. However, some manifestations emerge within the medium to long term and may lead to poor quality of life, death or both. These complications comprise severe pain (usually in the absence of anaesthetic agents), acute urinary retention (inability to voluntarily pass urine), vaginal cuts during sexual intercourse and haemorrhage (loss of blood) among other factors [2, 7, 8].

Owing to these severe repercussions and its illicit status in many countries, the WHO, human rights organisations and governments of most countries in SSA have garnered concerted efforts to end the practice. A prime legal obligation of sub-Saharan African countries is therefore to protect women and girls from FGM by instituting legislative measures to end the practice [9]. Consequently, there is an increased application of legal measures, which are common to most sub-Saharan African countries. Criminalisation of FGM in SSA is mostly in laws such as domestic violence acts, penal codes, women’s acts, among others [5, 9]. Sub-Saharan African countries with either of the aforementioned legislative measures include: Ghana (1994), Burkina Faso (1996), Ivory Coast (1998), Senegal (1999), and Togo (1998) [10]. Between 2007 and 2015, Zimbabwe, Uganda, South Sudan, Kenya, Guinea Bissau, Mozambique, The Gambia, and Cameroon also either introduced or amended existing laws to curb the practice [11]. Punishments have usually been fines or jail sentences [9].

Relative to sociocultural drivers, socioeconomic and demographic dimensions of FGM has been noted [12]. FGM serves as a social stratification mechanism whereby circumcised females are perceived to be in higher status and also constitutes prerequisite for inheritance in some practicing societies [13]. Some evidence indicate that disadvantaged socioeconomic position (usually measured by education and wealth) compels some women to admit the practice [14, 15]. Karmaker et al. [14] therefore proposed that future research must investigate varying rate of FGM by socioeconomic status in order to inform policy accordingly.

Investigation into FGM in Africa has been done in the past [16]. To the best of our knowledge, the association between socioeconomic status and demographich characteristics and FGM underpinned by recent evidence from national level surveys such as the Demographic and Health Survey has not been explored in SSA. This present study, therefore, seeks to examine the association between socioeconomic and demographic factors and FGM among women and female children in 12 selected countries in SSA. In addition to revealing the socioeconomic covariates of FGM in SSA, the outcome of the study would direct policy to socioeconomic driven priority populations within SSA who require anti FGM advocacy, education and public communication campaigns. These strategies will augment efforts of sub-Saharan African countries in their pursuit to achieve the Sustainable Development Goals (SDGs) 3 (ensure healthy lives and promote wellbeing for all at all age) and 5 which seeks to achieve gender equality and empower all women and girls everywhere [17].

## Methodology

### Data source

The study used pooled data from current Demographic and Health Surveys (DHS) conducted between January 1, 2010 and December 31, 2018 in 12 countries in SSA. The countries are Burkina Faso, Chad, Ethiopia, Guinea, Kenya, Mali, Niger, Nigeria, Senegal, Sierra Leone, Tanzania and Togo. These 12 countries were included in the study because their surveys had information on FGM and had questions on whether the woman herself had undergone FGM; and whether she had daughter(s) who have also undergone FGM. We excluded two countries (Côte d'Ivoire and Gambia) because although they had data on FGM, data for daughters of women aged 15–49 were non-existent. The 12 countries were considered to provide a holistic and in-depth evidence of FGM in SSA. DHS is a nationwide survey executed every five years across low-and-middle-income countries (LMICs). It is representative of each of these countries. Women’s files that have responses by women aged 15–49 were used in the study. The surveys targeted core maternal and child health indicators such as FGM, unintended pregnancy, contraceptive use, skilled birth attendance, immunisation among under-fives and intimate partner violence. Stratified dual-stage sampling approach was employed and the same questions were posed to women of all these countries and thus make it feasible for multi-country study. The study involved cluster sampling process (i.e. enumeration areas [EAs]), followed by systematic household sampling within the selected EAs. The sample frame usually excludes nomadic and institutional groups such as prisoners and hotel occupants. In this study, two different samples were considered. The first sample was made up of 130,605 women aged 15–49 who responded to questions on whether they had undergone FGM. The second sample was made up of 122,941 women aged 15–49 who had at least one daughter and responded to questions on whether their daughter(s) had undergone FGM. We followed the ‘Strengthening the Reporting of Observational Studies in Epidemiology’ (STROBE) statement in conducting this study.

### Study variables

#### Dependent variable

The dependent variable in this study was “has had FGM or undergone FGM’. To derive this variable, respondents were asked if their genital area was “nicked with nothing removed;” “something removed,” or “sewn shut”. The responses were ‘Yes’ and ‘No’. These were coded as follows; No = 0, Yes = 1. Respondents who had daughters were further asked how many of their daughter(s) had their genital area “nicked with nothing removed;” “something removed,” or “sewn shut”. The response ranged from ‘no daughter’ to ‘1, 2, 3, 4, 5, 6, 7 daughters’. To provide a binary outcome, women who said none of their daughters went through FGM were coded as ‘No = 0’ and those who had at least one daughter going through FGM were coded ‘Yes = 1’.

#### Explanatory variables

The main explanatory variable was ‘socio-economic status’. Following some previous studies [18–20], we used wealth quintile and maternal education as proxy measures of socio-economic status. In the standard DHS, wealth quintile is computed from data on household ownership of selected assets such as bicycle, materials used for house construction, television, type of water access and sanitation facilities. A composite variable, wealth status, is created from these assets through Principal Component Analysis (PCA) by placing households on a continuous measure of relative wealth after which households are categorized into five wealth quintiles namely poorest, poorer, middle, richer and richest [21]. Maternal education, on the other hand is a standardized variable of highest education attained and offers level of education in these four categories: No education, Primary, Secondary, and Higher [21]. We maintained the original categorization and coding of these two variables, (i. e. wealth quintile and maternal education). Apart from these independent variables, we controlled for country of survey and demographic variables like age, residence, marital status, occupation, frequency of reading newspaper, frequency of listening to radio and frequency of watching television. The coding of these variables are found in Table 1. Apart from country of survey, which was included a priori, selection of all the explanatory variables was influenced by previous studies [14, 22, 23] and their availability in the datasets.

**Table 1:** Socio-demographic characteristics of respondents (Weighted)

Variables	Women aged 15–49 (N = 130,605)		Women who had daughters (N = 122,941)	
	Frequency (n)	Percentage (%)	Frequency (n)	Percentage (%)
Wealth quintile				
Poorest	22,030	16.9	20,915	17.0
Poorer	22,711	17.4	21,583	17.6
Middle	24,397	18.7	23,188	18.9
Richer	27,590	21.1	26,081	21.2
Richest	33,877	25.9	31,174	25.4
Education				
No education	61,467	47.1	58,676	47.7
Primary	33,036	25.3	31.637	25.7
Secondary	29,870	22.9	26,649	21.7
Higher	6232	4.8	5979	4.9
Age				
15–19	25,612	19.6	21,820	17.8
20–24	23,301	17.8	21,585	17.6
25–29	23,598	18.1	22,508	18.3
30–34	19,075	14.6	18,598	15.1
35–39	16,575	12.7	16,272	13.2
40–44	12,168	9.3	12,010	9.8
45–49	10,276	7.9	10,148	8.25
Residence				
Urban	49,806	38.1	46,551	37.9
Rural	80,799	61.9	76,390	62.1
Marital status				
Single	30,612	23.4	26,313	21.4
Married	84,940	65.0	82.064	66.7
Cohabitation	6067	4.7	5877	4.8
Widowed/divorced/separated	8986	6.9	8,687	7.1
Occupation				
Not working	44,507	34.1	40,656	33.1
Managerial	4230	3.2	4102	3.3
Clerical	1202	0.9	1152	0.9
Sales	22,815	17.5	22,711	18.5
Agricultural	32,909	25.2	31,082	25.3
Household	4400	3.4	4349	3.5
Services	6864	5.3	6863	5.6
Manual	13,177	10.1	11,526	9.4
Other	501	0.4	500	0.4
Frequency of reading newspaper/magazine				
Not at all	105,754	81.0	99,485	80.9
Less than a week	14,091	10.8	13,540	11.0
At least once a week	10,760	8.2	9916	8.1
Frequency of listening to radio				
Not at all	44,339	34.0	41,947	34.1
Less than once a week	28,879	22.1	27,095	22.1
At least once a week	57,387	43.9	53,899	43.8
Frequency of watching television				
Not at all	71,830	55.0	66,491	54.1
Less than once a week	18,389	14.1	17,701	14.4
At least once a week	40,388	30.9	38,749	31.5

### Statistical analysis

**Fig. 1:**
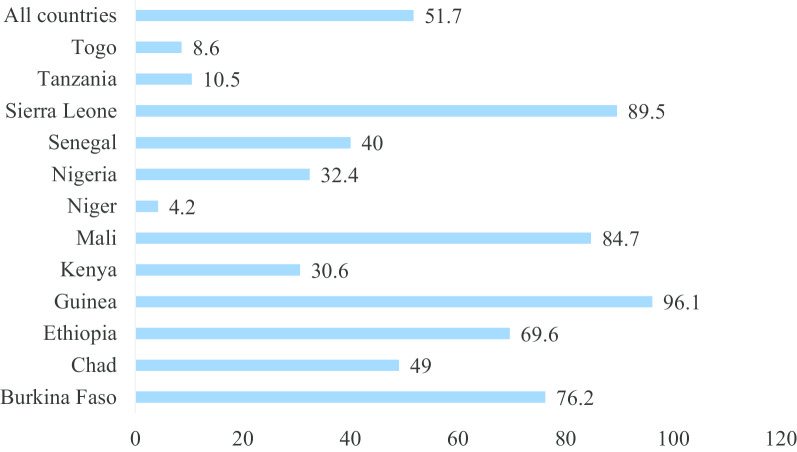
Proportion of women aged 15–49 who have undergone FGM in SSA

**Fig. 2:**
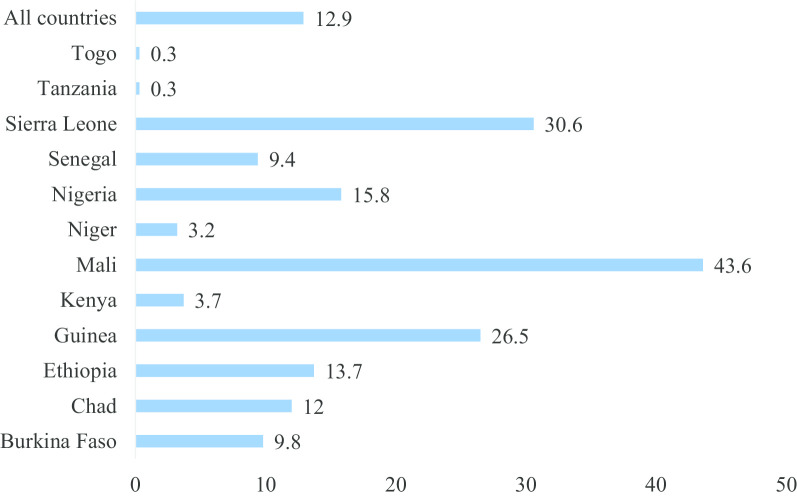
Proportion of daughters of women aged 15–49 who have undergone FGM in SSA

The analyses begun with computation of FGM among women aged 15–49 and their daughters. Secondly, we appended the datasets and this generated a total sample of 130,605 women aged 15–49 with data on FGM and 122,941 of women aged 15–49 who had at least one daughter and answered questions on FGM among their daughters. After appending, we presented the weighted socio-demographic characteristics of women aged 15–49 and those who had at least one daughter (see Table 1). After this, we calculated the prevalence of FGM among women aged 15–49 and their daughters and presented them using charts (see Figs. 1, 2). We also calculated the prevalence of FGM among women and their daughters across their socio-economic status and other socio-demographic characteristics. We presented these using proportions, chi-square and p values. Finally, two binary logistic regression models were built. The first model (Model I) reports on womens’ FGM whilst the second model (Model II) reports on daughters’ FGM.

The model fitness specification was done with the Hosmer–Lemeshow test while multicollinearity was checked using the variance inflation factor (VIF). The multicollinearity test for the explanatory variables for FGM among women (Mean VIF = 1.46, Max VIF = 1.90, Minimum = 1.06) and that of FGM among daughters (Mean VIF = 1.45, Max VIF = 1.80, Minimum = 1.06) showed no evidence of collinearity among the independent variables. Binary logistic regression was employed because our dependent variables were measured using a binary factor. Results for the regression analysis were presented as crude odds ratios (COR) and adjusted odds ratios (AOR), with their corresponding 95% confidence intervals (CI) signifying precision. The analyses were carried out with STATA version 13.0 with inherent sample weight applied. Sample weight was applied and the survey command (svy) was used to account for the complex sampling design of the survey.

### Ethical approval

The DHS surveys obtain ethical clearance from the Ethics Committee of ORC Macro Inc. as well as Ethics Boards of partner organisations of the various countries such the Ministries of Health. During each of the surveys, either written or verbal consent was provided by the women. Since the data was not collected by the authors of this manuscript, we sought permission from MEASURE DHS’s website and access to the data was provided after our intent for the request was assessed and approved on 3rd April, 2019. The dataset is freely available at https://dhsprogram.com/data/available-datasets.cfm.

## Results

### Descriptive results

Table 1 shows results on the sociodemographic characteristics of the respondents. On the participants who participated in our study, 25.9% were of the richest wealth quintile and 25.4% were those in the richest quintile who had at least a daughter. Similarly, 47.1% had no formal education and 47.7% indicated they had one or more daughters. With the age category of participants, 19.6% of women were aged 15–19 and 18.3% of women 25–29 had one or more daughters. Also, 62 out of 100 women who participated in the study lived in rural areas with 62.1% of women living in rural areas having one or more daughters. Further on marital status of respondents, 65% of the women were married and 66.7% of those who are married have at least one daughter. Again, the greatest proportion of the women who participated in the study were working (65.9%) and a corresponding 66.9% of those within this category had 1 or more daughters. On reading newspapers/magazine, majority of the women (81%) had not read newspapers/magazine within a week. In the same vein, 43.9% of the women listened to radio at least once a week. Finally, 55% of the women indicated they do not watch television at all and 54.1% of women who do not watch television at all had one or more daughters (see Table 1).

### Prevalence of FGM in SSA

Figure 1 and 2 are graphical presentations of the proportions of women and their daughters who had undergone FGM in SSA. The results indicated that overall, 51.7% of women and 12.9% of their daughters had undergone FGM in SSA. The highest proportion of FGM among women in SSA was in Guinea (96.1%) whiles the majority of FGM among daughters occurred in Mali (43.6%). Niger had the lowest prevalence of FGM among women (4.2%) while both Togo and Tanzania had the lowest proportions (0.3%) of FGM among daughters in SSA.

### Distribution of factors associated with FGM

Table 2 displays results of the prevalence of FGM across socio-demographic characteristics of women from 12 countries in SSA as well as the proportion of women indicating at least 1 of their daughters had undergone FGM. On wealth quintile of women who had undergone FGM, and whose daughters had also undergone FGM, 60.2% and 18.7% respectively, were in the poorest quintile. Besides, majority (65.8%) of women who had undergone FGM had no formal education and 20.8% of women in this category had at least 1 daughter undergone FGM. Also, 59.7% of women who had FGM were aged 45–49. Further, majority (54.9%) of women and a corresponding 15.4% of their daughters who had FGM were of rural background. On marital status, 57.4% of women who were in marriage had FGM and 17.6% indicated one or more of their daughters had also undergone FGM. In terms of occupation, women of other occupation (64%) and 16.9% who were in agriculture had undergone FGM. Again, 56.5% of women who did not read newspaper at all had undergone FGM and 15.1% of these women indicated one or more of their daughters were also subjected to FGM. Similarly, 58.6% and 59.3% of women who did not listen to radio at all nor watched television respectively had undergone FGM. The Chi-square analysis showed that all the independent variables were statistically significantly associated with FGM among women and their daughters in SSA at 95% level of significance.

**Table 2:** Distribution of factors associated with FGM in SSA

Variables	FGM among women			FGM among daughters		
	Yes	No	χ^2^ (P value)	Yes	No	χ^2^ (P value)
Wealth quintile			7.9 (< 0.001)			8.0 (< 0.001)
Poorest	60.2	39.8		18.7	81.3	
Poorer	54.9	45.1		15.1	84.9	
Middle	52.8	47.2		13.7	86.3	
Richer	50.5	49.5		11.7	88.3	
Richest	42.9	57.1		6.8	93.2	
Education			29.6 (< 0.001)			21.2 (< 0.001)
No education	65.8	34.2		20.8	79.2	
Primary	39.2	60.8		5.6	94.4	
Secondary	38.8	61.2		5.0	95.0	
Higher	32.8	67.2		3.5	96.5	
Age			973.3 (< 0.001)			21.5 (< 0.001)
15–19	46.0	54.0		1.2	98.8	
20–24	47.9	52.1		5.9	94.1	
25–29	51.9	48.5		11.7	88.3	
30–34	53.7	46.3		17.7	82.3	
35–39	55.9	44.1		21.4	78.6	
40–44	56.0	44.0		21.8	78.2	
45–49	59.7	40.4		22.7	77.3	
Residence			875.0 (< 0.001)			6.3 (< 0.001)
Urban	46.5	53.5		8.6	91.4	
Rural	54.9	45.1		15.4	84.6	
Marital status			12.5 (< 0.001)			18.0 (< 0.001)
Single	40.7	59.4		0.5	99.5	
Married	57.4	42.6		17.6	82.4	
Cohabitation	31.5	68.5		3.3	96.7	
Widowed/divorced/separated	47.1	52.9		10.9	89.1	
Occupation			12.3 (< 0.001)			6.8 (< 0.001)
Not working	46.9	53.1		10.5	89.5	
Managerial	35.7	64.3		6.6	93.4	
Clerical	35.1	64.9		5.0	95.0	
Sales	49.7	50.3		15.3	84.7	
Agricultural	63.0	37.0		16.9	82.1	
Household	27.4	72.6		2.6	97.4	
Services	41.1	58.9		9.8	90.2	
Manual	61.3	38.7		14.7	85.3	
Other	64.0	36.0		7.3	92.7	
Frequency of reading newspaper			17.7 (< 0.001)			9.5 (< 0.001)
Not at all	56.5	43.5		15.1	84.9	
Less than a week	30.1	69.9		3.0	97.0	
At least once a week	29.9	70.1		2.4	97.6	
Frequency of listening to radio			7.6 (< 0.001)			832.3 (< 0.001)
Not at all	58.6	41.4		16.5	83.5	
Less than once a week	52.7	47.3		12.6	87.4	
At least once a week	45.7	54.3		10.2	89.8	
Frequency of watching television			14.4 (< 0.001)			8.0 (< 0.001)
Not at all	59.3	40.7		16.3	83.7	
Less than once a week	46.9	53.1		10.9	89.1	
At least once a week	39.2	60.8		7.4	92.6	

### Binary logistic regression results of the influence of socio-economic status and other socio-demographic characteristics on FGM

Table 3 presents the results for the binary logistic regression analysis on the influence of socio-economic status and other socio-demographic characteristics on FGM in SSA. The results show that FGM among women and their daughters decreases with wealth status, with women within the richest wealth quintile (AOR = 0.51, CI 0.48–0.55) and their daughters (AOR = 0.64, CI 0.59–0.70) being less likely to undergo FGM compared to those within the poorest wealth quintile. Across education, the odds of women and their daughters undergoing FGM decreased with increasing level of education as women with higher level of education had the lowest propensity of undergoing FGM (AOR = 0.62, CI 0.57–0.68) as well as their daughters (AOR = 0.32, CI 0.24–0.38). Compared with women from Burkina Faso, women from Guinea, (AOR = 8.51, CI 7.64–9.49) had higher odds of undergoing FGM whiles those from Mali were more likely to have daughters who had undergone FGM (AOR = 12.47, CI 11.36–13.70). The likelihood of FGM among women and their daughters increased with age, with women aged 45–49 (AOR = 1.85, CI 1.73–1.99) and their daughters (AOR = 12.61, CI 10.86–14.64) more likely to undergo FGM. While women in rural areas were less likely to undergo FGM (AOR = 0.81, CI 0.78–0.84), their daughters were more likely to undergo FGM (AOR = 1.09, CI 1.03–1.15). Married women (AOR = 1.67, CI 1.59–1.75) and their daughters (AOR = 8.24, CI 6.88–9.87) had the highest odds of undergoing FGM. Furthermore, we found that women whose occupation was in the agricultural sector had high likelihood of undergoing FGM (AOR = 1.24, CI 1.19–1.29) while those in the manual sector were more likely to have daughters who had undergone FGM (AOR = 1.09, CI 1.02–1.18). Women who had exposure to media (newspaper/magazine, radio and television) were less likely to undergo FGM and had daughters with FGM.

**Table 3:** Results of binary logistic regression analysis of the influence of socio-economic status and other socio-demographic characteristics on FGM

Variables	FGM among women	FGM among daughters
	Model I AOR (95%CI)	Model I AOR (95%CI)
Socio-economic variables		
Wealth quintile		
Poorest	Ref	Ref
Poorer	0.76*** (0.72–0.79)	0.81*** (0.76–0.85)
Middle	0.74***(0.70–0.77)	0.79***(0.75–0.84)
Richer	0.65***(0.62–0.69)	0.76***(0.71–0.80)
Richest	0.51***(0.48–0.55)	0.64***(0.59–0.70)
Education		
No education	Ref	Ref
Primary	0.80***(0.77–0.84)	0.57***(0.53–0.60)
Secondary	0.77***(0.73–0.80)	0.61***(0.56–0.65)
Higher	0.62***(0.57–0.68)	0.32***(0.24–0.38)
Socio-demographic variables		
Age		
15–19	Ref	Ref
20–24	1.11***(1.06–1.17)	3.24***(2.78–3.75)
25–29	1.21***(1.14–1.28)	5.76***(4.98–6.66)
30–34	1.34***(1.26–1.42)	9.49***(8.20–10.97)
35–39	1.48***(1.39–1.58)	11.83***(10.23–13.69)
40–44	1.60***(1.50–1.71)	12.41***(10.71–14.39)
45–49	1.85***(1.73–1.99)	12.61***(10.86–14.64)
Residence		
Urban	Ref	Ref
Rural	0.81***(0.78–0.84)	1.09**(1.03–1.15)
Marital status		
Single	Ref	Ref
Married	1.67***(1.59–1.75)	8.24***(6.88–9.87)
Cohabitation	1.06 (0.97–1.15)	3.24***(2.78–4.49)
Widowed/divorced/separated	1.50***(1.40–1.61)	5.81***(4.78–7.05)
Occupation		
Not working	Ref	Ref
Managerial	1.13**(1.04–0.23)	1.09(0.93–1.27)
Clerical	0.97(0.83–1.13)	0.83(0.62–1.11)
Sales	1.07**(1.03–1.12)	1.02(0.99–1.08)
Agricultural	1.24***(1.19–1.29)	0.99(0.93–1.08)
Household	084***0.78–0.92)	0.65***(0.53–0.81)
Services	0.96(0.89–1.03)	0.85**(0.77–0.95)
Manual	1.11***(1.04–1.17)	1.09* (1.02–1.18)
Other	0.91(0.74–1.12)	0.61*(0.42–0.90)
Frequency of reading newspaper/magazine		
Not at all	Ref	Ref
Less than a week	0.83***(0.79–0.88)	0.72***(0.64–0.81)
At least once a week	0.66***(0.62–0.70)	0.61*** (0.52–0.71)
Frequency of listening to radio		
Not at all	Ref	Ref
Less than once a week	1.01(0.96–1.05)	1.00(0.95–1.06)
At least once a week	0.92***(0.88–0.95)	0.99(0.94–1.04)
Frequency of watching television		
Not at all	Ref	Ref
Less than once a week	1.02(0.97–1.07)	0.93*(0.87–0.99)
At least once a week	0.94*(0.90–0.99)	0.81***(0.75–0.86)
Survey country		
Burkina Faso	Ref	Ref
Ethiopia	0.89***(0.83–0.95)	2.06***(1.87–2.26)
Guinea	8.51***(7.64–9.49)	4.20***(3.90–4.52)
Kenya	0.17***(0.16–0.18)	0.65*** (0.58–0.73)
Mali	1.97***(1.79–2.16)	12.47***(11.36–13.70)
Nigeria	0.15***(0.14–0.16)	2.49***(2.30–2.69)
Niger	0.01***(0.01–0.02)	0.24***(0.20–0.30)
Sierra Leone	3.26***(3.06–3.18)	3.35***(3.12–3.60)
Sengal	0.21***(0.20–0.23)	1.22***(1.11–1.33)
Chad	0.29***(0.27–0.31)	1.37***(1.25–1.50)
Togo	0.03***(0.02–0.03)	0.04***(0.03–0.06)
Tanzania	0.04***(0.04–0.05)	0.06***(0.04–0.08)
Pseudo R^2^	0.336	0.269

Exponentiated coefficients; 95% confidence intervals in brackets

*Ref *reference category, *COR* crude odds ratio, *AOR *adjusted odds ratio

*p < 0.05, **p < 0.01, ***p < 0.001

## Discussion

The current study provides a distinctive opening by examining the geographical variations of FGM prevalence in 12 selected SSA countries where the act is generally predominant. The strength of the study is that by investigating the geographical distributions of FGM in the midst of socio-cultural determinants across these countries, planned interventions may not focus on a “one size fits all” approach to alleviate the current burden. The overall prevalence of FGM in the selected SSA countries was 51.7% (women) and 12.9% (daughters of circumcised women) using the most recent survey, the 2010–2018 DHS. However, there were significant geographical variations between countries. For instance, Guinea had the highest proportion of FGM (96.1%) while the majority of FGM among daughters occurred in Mali (43.6%). Niger had the lowest prevalence of FGM among women (4.2%) whiles both Togo and Tanzania had the lowest proportions (< 1%) of FGM among daughters in the selected SSA (see Figs. 1, 2).

As with previous research [14, 23–27], the current study revealed that wealth index, maternal educational status, age of mothers, place of residence (urban versus rural), marital status, occupational status, and media exposure were factors associated with maternal and/or daughters’ FGM experience. Specifically, FGM among women and their daughters decreased with wealth index, with women within the richest wealth quintile and their daughters less likely to undergo FGM compared to those within the poorest wealth quintile. This finding implies that the risk of FGM is primarily higher in very poor households than in rich households and that better socio-economic status (wealth) is a factor inversely associated with FGM practice among women and their daughters. While wealth is usually connected with other social parameters (e.g., place of residence and/or household level of education), it undoubtedly remains associated with decreased risk of FGM in some countries [25]. Literature to date has shown that affluent women have strong decision-making power on harmful traditional practices like FGM on themselves and their daughters because of their wealth status [23]. Similar to other studies [28, 29], the odds of women and their daughters undergoing FGM decreased with increasing level of education as women with higher level of education had the lowest propensity of undergoing FGM as well as their daughters. Therefore, women's schooling could be linked with a decline in FGM across studied countries noted for the exercise, though with varying degrees. The FGM risk for educated women and their daughters is lower than their counterparts with no formal education [25]. By making inference, women with no formal education have higher odds of possibly circumcising their daughters in the future compared to those with varying degrees of education. Educationally empowered women are more likely to turn down any societal pressure to circumcise their daughters [29]. Using the 2000 Egypt Demographic and Health Survey data, Afifi [28] noted that women with more empowerment and educated women were 8.06 times less likely to encourage FGM for their daughters compared other women with less empowerment and low level of education. According to Andro et al. [25], educational status should not be deduced as a direct causal explanatory factor for FGM (i.e., may occur before schooling) because women do not control the practice, but may work as a proxy that is influenced by family background. Hence, investing in schooling, including their daughters, might relate to more openness about criticisms against this practice and a better awareness of its adverse effects. UNICEF [5] reiterates that the percentage of daughters who experience FGM reduces as their mother's level of education increases.

Whereas education appears to be an important factor, current results also show that other connecting socio-demographic factors, such as place of residence and age status, also play a crucial role towards FGM experience. Surprisingly, while women in rural areas were less likely to undergo FGM, their daughters were more likely to experience the practice. This finding contradicts the commonly held assumption that the risk of FGM is always higher in rural than in urban areas [25]. It is possible that women's residential status at the time of the DHS survey was not a fair reflection of their geographical origin. Rural–urban migration is high in many SSA Africa [30]. For many women who migrate to urban centres, unfulfilled expectations and aspirations may force them to revert to their rural communities. Despite their unpleasant urban experiences, this transition regardless of how those experiences lasted, may present these women with considerable exposure and enlightenment about harmful practices such as FGM (see convention theory, [31, 32]). Such women may resist any attempt or social pressure to undergo the mutilation exercise or have their daughters undergo the practice. For countries (e.g., Guinea, Sierra Leone, Mali, Burkina Faso), with high prevalence of FGM, it is also possible that strictly upholding to cultural norms and values may strongly influence women to undergo FGM while residing in urban areas. Hicks [33] has emphasized that continuous practice of FGM in some urban settlements is because of continued socioeconomic ties with rural settings. For daughters who live in rural areas, the maintenance of FGM hinges on a conventional norm of marriageability which is universal among some intermarrying groups in SSA and forces them to continuously adhere to the practice. For most patriarchal systems, daughters of women from these groups who support FGM are caught up in this self-reinforcing "belief” trap (e.g., loss of family honor, unmarriageable daughter [31, 32]).

Further, the likelihood of FGM among women and their daughters increased with age, with women aged 45–49 and their daughters more likely to undergo FGM. Perhaps, older women and daughters are more entrenched with the conventional FGM practice because they are quite accustomed to traditional practices that typify group identity and culture, hence may strongly uphold traditions of marriage, family, and other gender role orientations [34]. Both older women and their daughters may have been strongly influenced by living with their elderly and grandparents while learning about issues related to their culture and the need to uphold them through vicarious experiences over time regardless of how others perceive those practices (e.g., FGM). For societies with high prevalence, FGM is seen as rite of passage to womanhood with strong ancestral and sociocultural roots [35]. With the current modernization, it is very possible that entrenched cultural norms will decrease the practice of FGM, where women and their daughters of the younger generation will not be willing to undergo FGM.

The present study also confirmed that married women and daughters had the highest odds of undergoing FGM. This is because the autonomy and economic independence of many women and their daughters are solely driven by men in most SSA societies; hence marriage might be seen as the most probable means of maintaining a living by women who are married. By economically benefitting from their marriage homes with FGM as a prerequisite, daughters are encouraged to undergo FGM because of the possibility of attracting potential suitors (i.e., future husbands, [36, 37]). Different studies have already shown that women who are circumcised are believed to be better candidates for marriage [38, 39].

Again, women whose occupation was in the agricultural sector had high likelihood of undergoing FGM whiles those in the manual sector were more likely to have daughters who had undergone FGM. Significantly, it is worth noting that the main types of employment in identified countries (e. g., Guinea, Sierra Leone, Mali, Burkina Faso, Ethiopia, Chad) with high FGM prevalence are in the informal sector (e.g., subsistence farming, fishing and manual work) (e.g., hawking on the streets, petty trading, and other manual labour [40]). Such jobs do not necessarily require a higher educational certification or formal education and skilled labour, and are quite often less paid. Mothers and daughters with these low paying jobs in such societies are not economically empowered enough to make major household decisions, including entrenched traditions and cultural norms such as FGM [40]. Women who had exposure to media (newspaper/magazine, radio and television) were less likely to undergo FGM and have daughters who had undergone FGM. Exposure to mass media (e.g., newspapers, television, or radio) decreases the likelihood of FGM. It is reasonable to say that media exposure might have a similar influence as educational attainment [39]. Some previous studies [34, 41] in Nigeria have demonstrated that being exposed to mass media campaigns had a positive impact on changing attitudes and promotion for the discontinuation of FGM.

### Strengths and limitations

Scholarly information to date on FGM has predominantly been limited to ethnographic, small-scale and community-based assessments. Therefore, the use of different large-scale national surveys from 12 SSA countries to investigate FGM prevalence, its pervasiveness and identified factors across women and their daughters would provide a sharper focus in tailoring appropriate interventions to manage this cultural practice. The geographical distributions of FGM would help adopt context specific strategies and not one size fit all approach and further guide nationwide government programming decisions. Despite the strengths, some limitations inherent in the current study cannot be underestimated. First, the DHS data used were cross-sectional in nature, meaning that inferences drawn from the findings to represent causality should be interpreted with caution. However, scholarly support from previous research and theory that led the current study provides backing to the drawn interpretations. Future studies should target longitudinal designs to provide in-depth sub-regional patterns through time series analysis. Second, the use of secondary data further limits the scope of this current study to measured variables within the dataset and compared to most DHS, the lack of qualitative data restricts in-depth understanding and explanation of patterns usually characterized with quantitative analysis. Third, collecting self-reported data on sensitive issue like FGM is somewhat difficult because measures could be very private, intimate and often connected to self-image and personality. Therefore, the possibility of under or over-reporting, memory distortions and other social desirability issues could be high, which may have blown-up the noted associations between measured variables. Fourth, considerations were not given to varying socio-cultural diversity (e.g., ethnicity) and other sub-regional factors such as country-specific norms, beliefs and cultural values which influence FGM practice. Future research could consider these variables to unearth a clearer understanding about the continuation and abolishing of this cultural issue. Finally, the differences in survey years can limit the comparability of the findings since modernization may limit the practice of FGM in more current surveys compared to older ones. However, all the surveys fall within the same DHS wave and therefore analogous.

### Practical implications

Current findings identified relevant socioeconomic, demographic factors and regional differences regarding FGM practice in 12 selected SSA countries. The geographic variation associated with factors facilitating FGM prevalence on women and their daughters can be used to plan for the distribution of the practice across the sub-continental states and even within specific countries identified as the hotspots. The practice of FGM depicts clear regional patterns, with West–East Africa noted with more concentration (i.e., high prevalence rates) in Guinea, Sierra Leone, Mali, Burkina Faso, Ethiopia, Chad, Senegal, Nigeria, and Kenya. Women and daughters overall FGM prevalence in the selected countries in SSA were approximate 52% and 13% respectively, though with varying low to high proportions, 4–96% for women and < 1 to 44% for daughters. Therefore, planned interventions to battle the FGM practice in these countries should be country specific because of their heterogonous socio-cultural diversity, though some countries may have similar characteristics (i.e., share certain social and historical ties (e.g., Burkina Faso-Mali; Ethiopia-Kenya). Specific interventions should focus more at the national level devoid of “one size fits all” approach often noted with public health interventions [16]. Significant efforts in these regional states will considerably reduce FGM overall national prevalence. Additional research focusing on individual, social and structural factors affecting FGM practice in these countries is urgently required. Studies could also compare variations in legal enforcement and adopted interventions within high and low prevalence countries [23].

## Conclusions

Within SSA the FGM practice covers wide geographical boundaries, from West to East Africa, with prevalence high in considerable areas of West Africa (Guinea, Sierra Leone, Mali, and Burkina Faso) and sections of East Africa (Ethiopia, Kenya, Tanzania). Women and daughters overall FGM prevalence in the selected SSA countries were approximately 52% and 13% respectively, though with low to high proportions, 4–96% for women and < 1 to 44% for daughters respectively. The current study revealed that wealth index, maternal educational status, age of mothers, place of residence (urban versus rural), marital status, occupational status, and media exposure were factors associated with maternal and/or daughters’ FGM experience. Specifically, women and daughters FGM experience were clustered among those in the poorest wealth quintile, less educated or no formal education (i.e., illiterate), older, urban areas, married, agriculture and manual (i.e., informal related) jobs, and less exposed to the media. Multifaceted interventions should include considerable efforts directed on advocacy and educational strategies like focus group discussions, peer teaching, mentor–mentee programmes at both national and community levels in regions noted with the FGM practice. Other legislative instruments, women capacity-building (e.g., entrepreneurial training), media advocacy and community dialogue could help address the FGM public health challenge. Future studies could consider the determinants related to intentions to continue the practice and its abandonment using more accurate measures in identified countries with low to high prevalence.

## Data Availability

Data for this study were sourced from Demographic and Health surveys (DHS) and available here: https://dhsprogram.com/data/available-datasets.cfm

## Abbreviations

SDG: Sustainable development goals
FGC: Female genital cutting
FC: Female circumcision
FGM: Female genital mutilation
SSA: Sub-Saharan Africa
DHS: Demographic and health surveys
COR: Crude odds ratios
AOR: Adjusted odds ratio
CI: Confidence interval
WHO: World Health Organization
ICF: Inner City Fund
USAID: United States Agency for International Development
UNDP: United Nations Development Programme
PCA: Principal component analysis
